# Supplementary material for: Qualitative rather than quantitative phosphoregulation shapes the end of meiosis I in budding yeast
Source: EMBO J. 2024 Feb 6;43(7):10. doi: 10.1038/s44318-024-00032-5 (PMC10987528; doi:10.1038/s44318-024-00032-5)
Supplement: Supplementary file 1 — Appendix [file 44318_2024_32_MOESM1_ESM.pdf]

Appendix

Appendix Figure S1: The phosphorylation landscape of the MI-MII transition in budding yeast -repeat 2 (page 1)

Appendix Figure S1 legend (page 2)

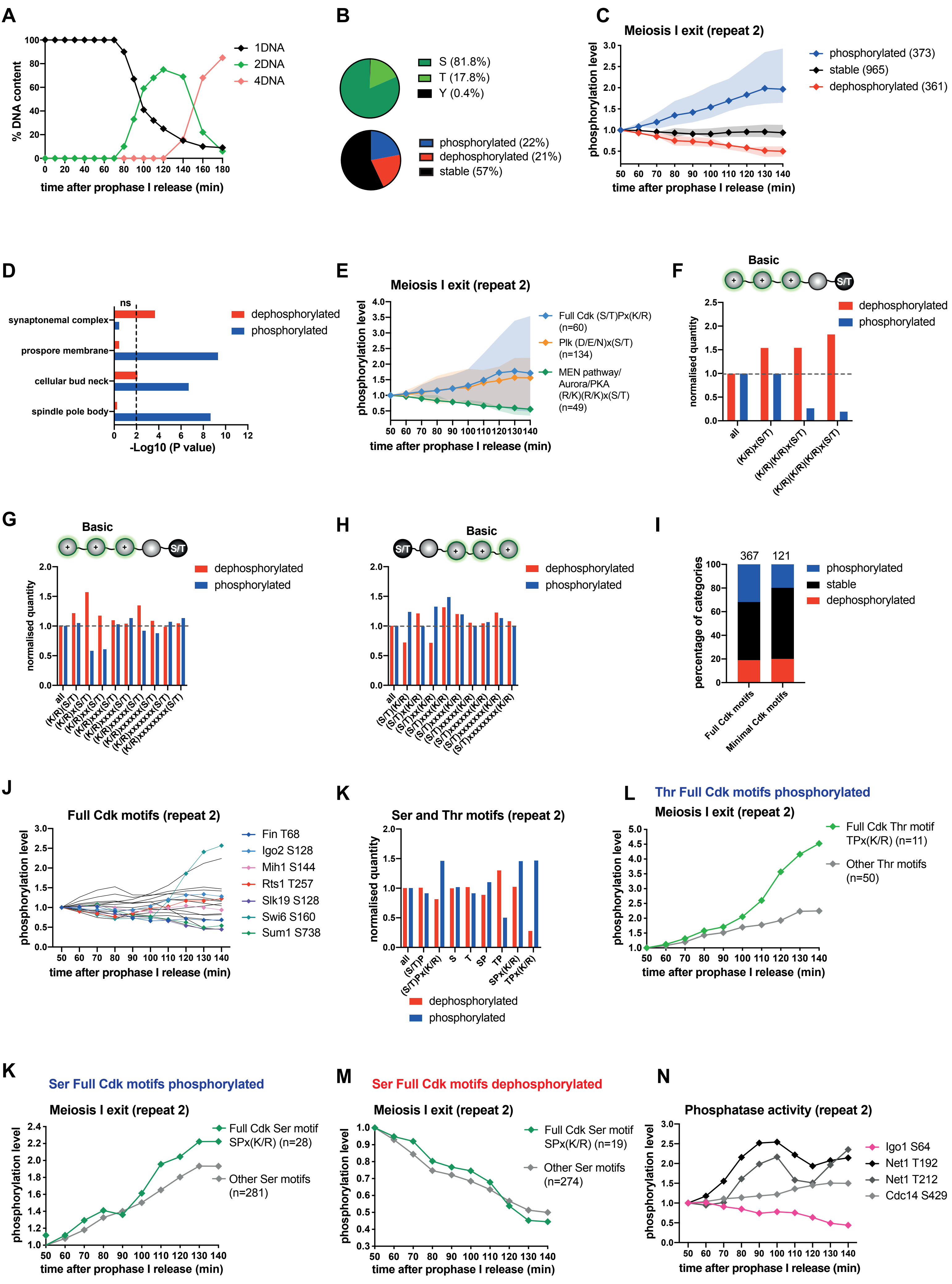
A
% DNA content
time after prophase I release (min)
1DNA
2DNA
4DNA
B
S (81.8%)
T (17.8%)
Y (0.4%)
phosphorylated (22%)
dephosphorylated (21%)
stable (57%)
C
Meiosis I exit (repeat 2)
phosphorylation level
time after prophase I release (min)
phosphorylated (373)
stable (965)
dephosphorylated (361)
D
ns
synaptonemal complex
prospore membrane
cellular bud neck
spindle pole body
-Log10 (P value)
dephosphorylated
phosphorylated
E
Meiosis I exit (repeat 2)
phosphorylation level
time after prophase I release (min)
Full Cdk (S/T)Px(K/R) (n=60)
Plk (D/E/N)x(S/T) (n=134)
MEN pathway/ Aurora/PKA (R/K)(R/K)x(S/T) (n=49)
F
Basic
S/T
normalised quantity
dephosphorylated
phosphorylated
all
(K/R)x(S/T)
(K/R)(K/R)x(S/T)
(K/R)(K/R)(K/R)x(S/T)
G
Basic
S/T
normalised quantity
dephosphorylated
phosphorylated
all
(K/R)(S/T)
(K/R)x(S/T)
(K/R)xx(S/T)
(K/R)xxx(S/T)
(K/R)xxxx(S/T)
(K/R)xxxxx(S/T)
(K/R)xxxxxx(S/T)
(K/R)xxxxxxx(S/T)
(K/R)xxxxxxxx(S/T)
H
Basic
S/T
dephosphorylated
phosphorylated
all
(S/T)(K/R)
(S/T)x(K/R)
(S/T)xx(K/R)
(S/T)xxx(K/R)
(S/T)xxxx(K/R)
(S/T)xxxxx(K/R)
(S/T)xxxxxx(K/R)
(S/T)xxxxxxx(K/R)
(S/T)xxxxxxxx(K/R)
I
367
121
percentage of categories
phosphorylated
stable
dephosphorylated
Full Cdk motifs
Minimal Cdk motifs
J
Full Cdk motifs (repeat 2)
phosphorylation level
time after prophase I release (min)
Fin T68
Igo2 S128
Mih1 S144
Rts1 T257
Slk19 S128
Swi6 S160
Sum1 S738
K
Ser and Thr motifs (repeat 2)
normalised quantity
all
(S/T)P
(S/T)Px(K/R)
S
T
SP
TP
SPx(K/R)
TPx(K/R)
dephosphorylated
phosphorylated
L
Thr Full Cdk motifs phosphorylated
Meiosis I exit (repeat 2)
phosphorylation level
time after prophase I release (min)
Full Cdk Thr motif TPx(K/R) (n=11)
Other Thr motifs (n=50)
K
Ser Full Cdk motifs phosphorylated
Meiosis I exit (repeat 2)
phosphorylation level
time after prophase I release (min)
Full Cdk Ser motif SPx(K/R) (n=28)
Other Ser motifs (n=281)
M
Ser Full Cdk motifs dephosphorylated
Meiosis I exit (repeat 2)
phosphorylation level
time after prophase I release (min)
Full Cdk Ser motif SPx(K/R) (n=19)
Other Ser motifs (n=274)
N
Phosphatase activity (repeat 2)
phosphorylation level
time after prophase I release (min)
Igo1 S64
Net1 T192
Net1 T212
Cdc14 S429

Appendix S1

The phosphorylation landscape of the MI-MII transition in budding yeast - repeat 2

A) One hundred cells were scored for spindle length and DNA content at each timepoint (0 to 180min after β-estradiol addition) to determine the cell cycle phases. 1DNA, 2DNA and 4DNA indicate the number of DNA mass observed by DAPI staining.

B) Fractions of quantified serine (S), threonine (T), or tyrosine (Y) phosphosites (top).

Fractions of phosphosites phosphorylated (blue), dephosphorylated (red), or remaining stable (black) through meiosis I exit (bottom).

C) Normalized median intensity profiles and interquartile range of the phosphosites that undergo a 1.5-fold decrease (red) or a 1.5-fold increase (blue) in phosphorylation abundance through meiosis I exit. Normalized median intensity profiles and interquartile range of the phosphosites remaining stable are in black.

D) Enrichment analysis of gene ontology cellular compartment (GOCC). -Log10 raw p-value is represented for the dephosphorylated proteins (red) and the phosphorylated proteins (blue) in the meiosis I exit datasets. Non-significant (ns) enrichment is represented on the left of the dashed line and significant enrichment on the right ($P < 0.05$).

E) Normalized median intensity profiles and interquartile range of the phosphosites that adhere to the three indicated kinase consensus motifs. For better visualization of the data, stable phosphosites of each category were not included in the median intensity profile. Of note (D/E/N)x(S/T) and (R/K)(R/K)x(S/T) categories exclude Cdk sites (S/T)P.

F-H) Percentage of dephosphorylated and phosphorylated sites categorized by amino acid motif identity after normalization to the total amount of sites. Phosphosites with single, double, or triple basic upstream amino acids in F, with distinct basic upstream amino acids are presented in G and with distinct basic downstream amino acids in H.

I) Percentage of the full Cdk sites (S/T)Px(K/R) and minimal Cdk sites (S/T)P in each category.

J) Profile plot of the 20 full Cdk sites in common between the meiosis I exit dataset repeat 1 that are dephosphorylated during mitotic exit (Figure 3D). Profiles of essential cell cycle regulators are highlighted in colors.

K) Percentage of dephosphorylated (red) and phosphorylated (blue) sites categorized by amino acid motif identity after normalization to total amount of sites.

L) Normalized median intensity profiles of sites phosphorylated on the full Cdk threonine motifs TPx(K/R) (light green) or the other threonine sites (grey) during meiosis I exit.

M) Normalized median intensity profiles of sites phosphorylated on the full Cdk serine motifs SPx(K/R) (dark green) or the other serine sites (grey) during meiosis I exit.

N) Normalized median intensity profiles of the sites dephosphorylated on full Cdk serine motifs SPx(K/R) (dark green) or the other serine sites (grey) during meiosis I exit.

O) Profile plot of phosphosites implicated in phosphatase regulation during meiosis I exit.
