# Supplementary figures and images for: Qualitative rather than quantitative phosphoregulation shapes the end of meiosis I in budding yeast
Source: EMBO J. 2024 Feb 6;43(7):10. doi: 10.1038/s44318-024-00032-5 (PMC10987528; doi:10.1038/s44318-024-00032-5)

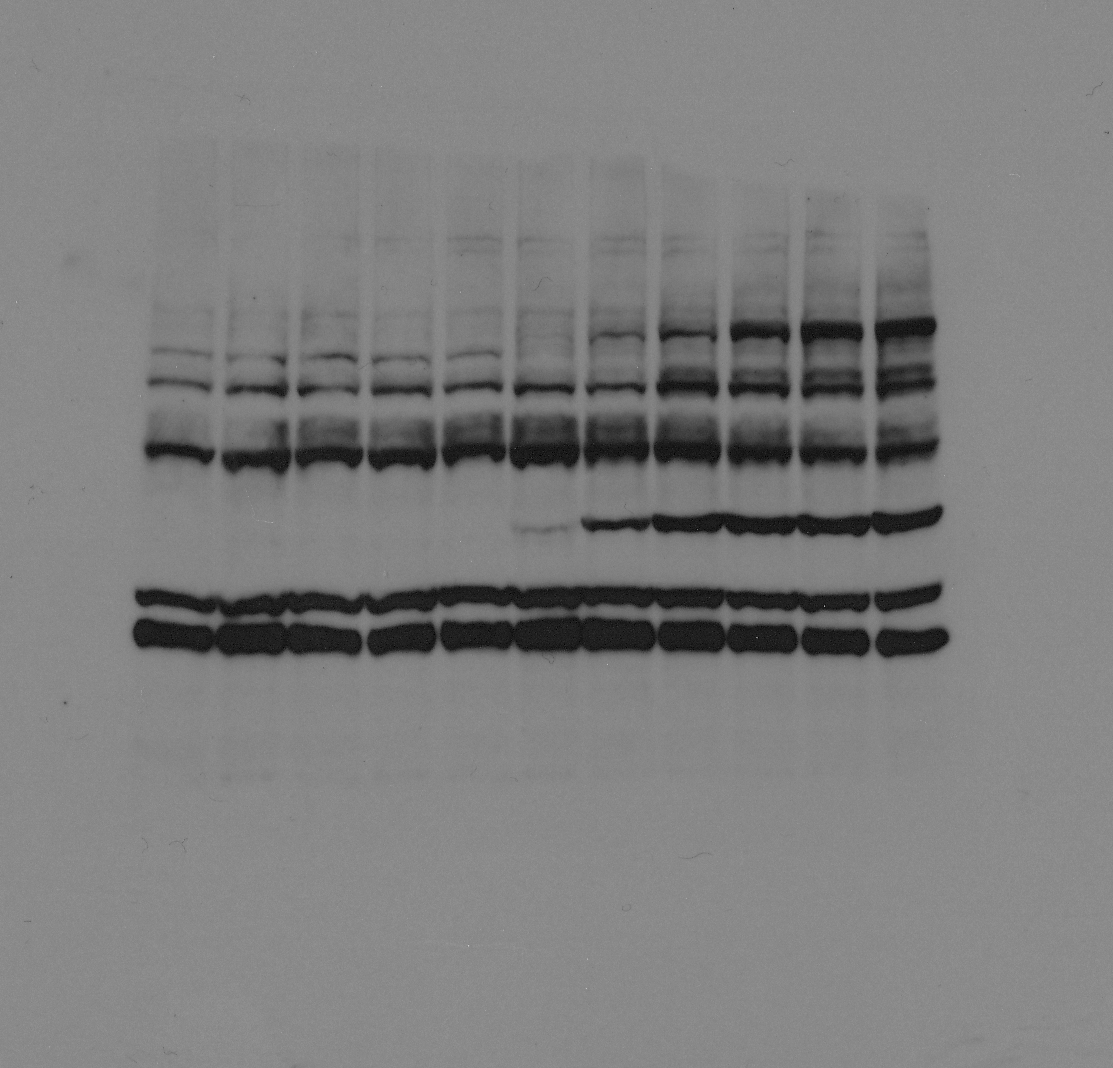

Supplement: Supplementary file 6 — Source Data Fig. 4 [file 44318_2024_32_MOESM6_ESM.zip › Figure 4/Figure 4F Image Data Blot/4F top.tif]

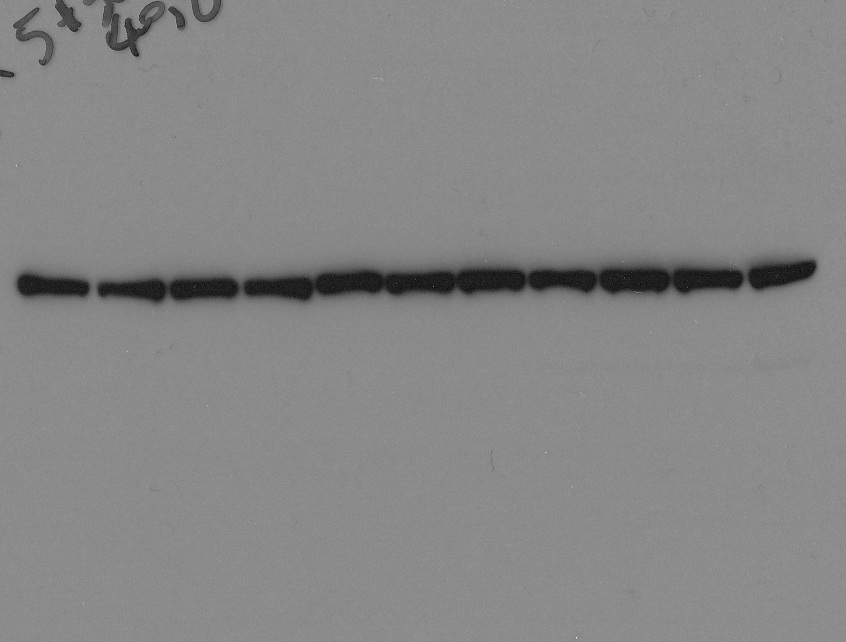

Supplement: Supplementary file 6 — Source Data Fig. 4 [file 44318_2024_32_MOESM6_ESM.zip › Figure 4/Figure 4F Image Data Blot/4F tubulin.tif]

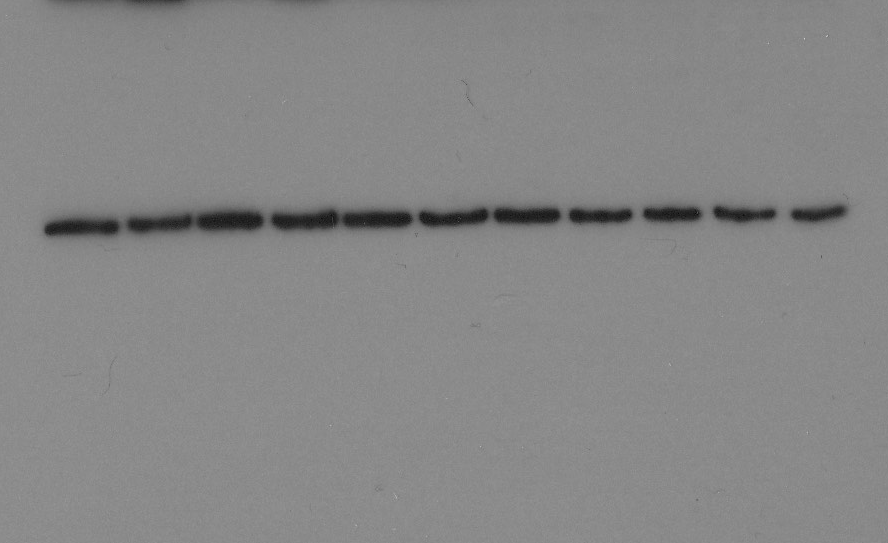

Supplement: Supplementary file 6 — Source Data Fig. 4 [file 44318_2024_32_MOESM6_ESM.zip › Figure 4/Figure 4C Image Data Blot/4C tubulin .tif]

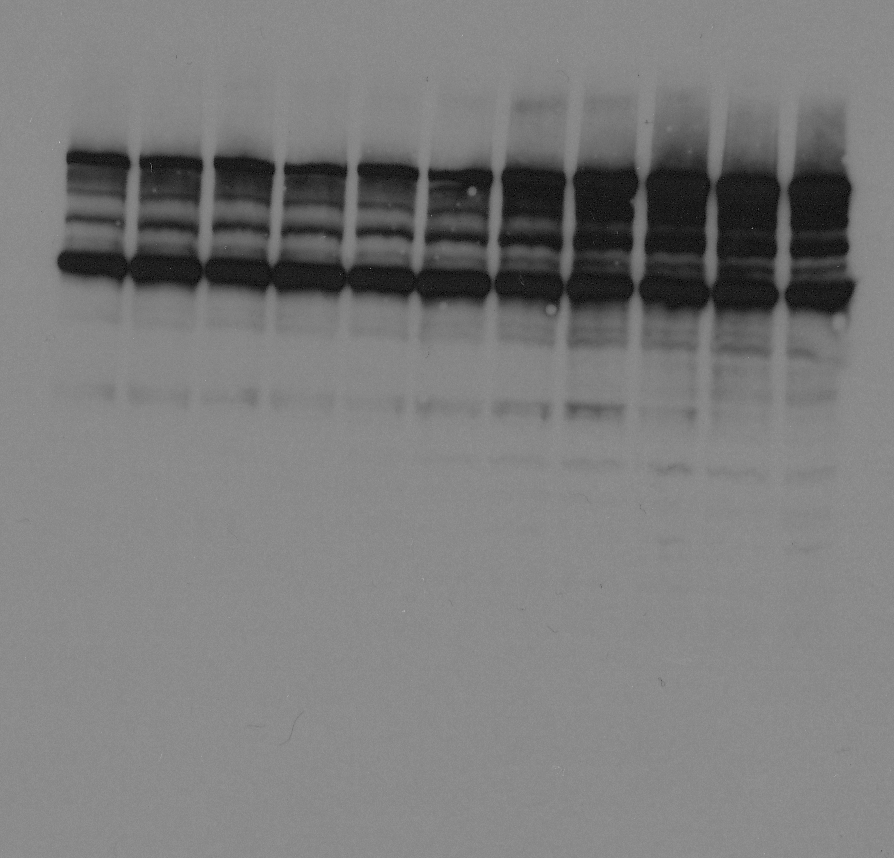

Supplement: Supplementary file 6 — Source Data Fig. 4 [file 44318_2024_32_MOESM6_ESM.zip › Figure 4/Figure 4C Image Data Blot/4C top .tif]

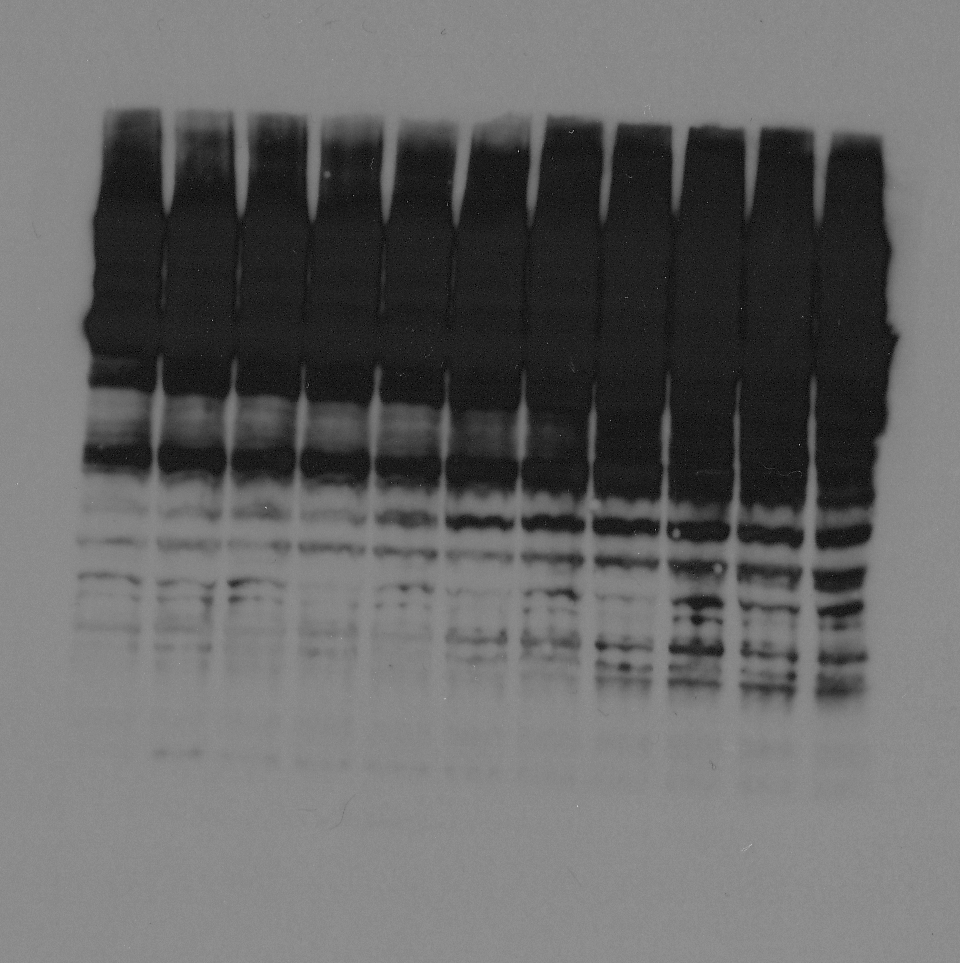

Supplement: Supplementary file 6 — Source Data Fig. 4 [file 44318_2024_32_MOESM6_ESM.zip › Figure 4/Figure 4C Image Data Blot/4C bottom .tif]

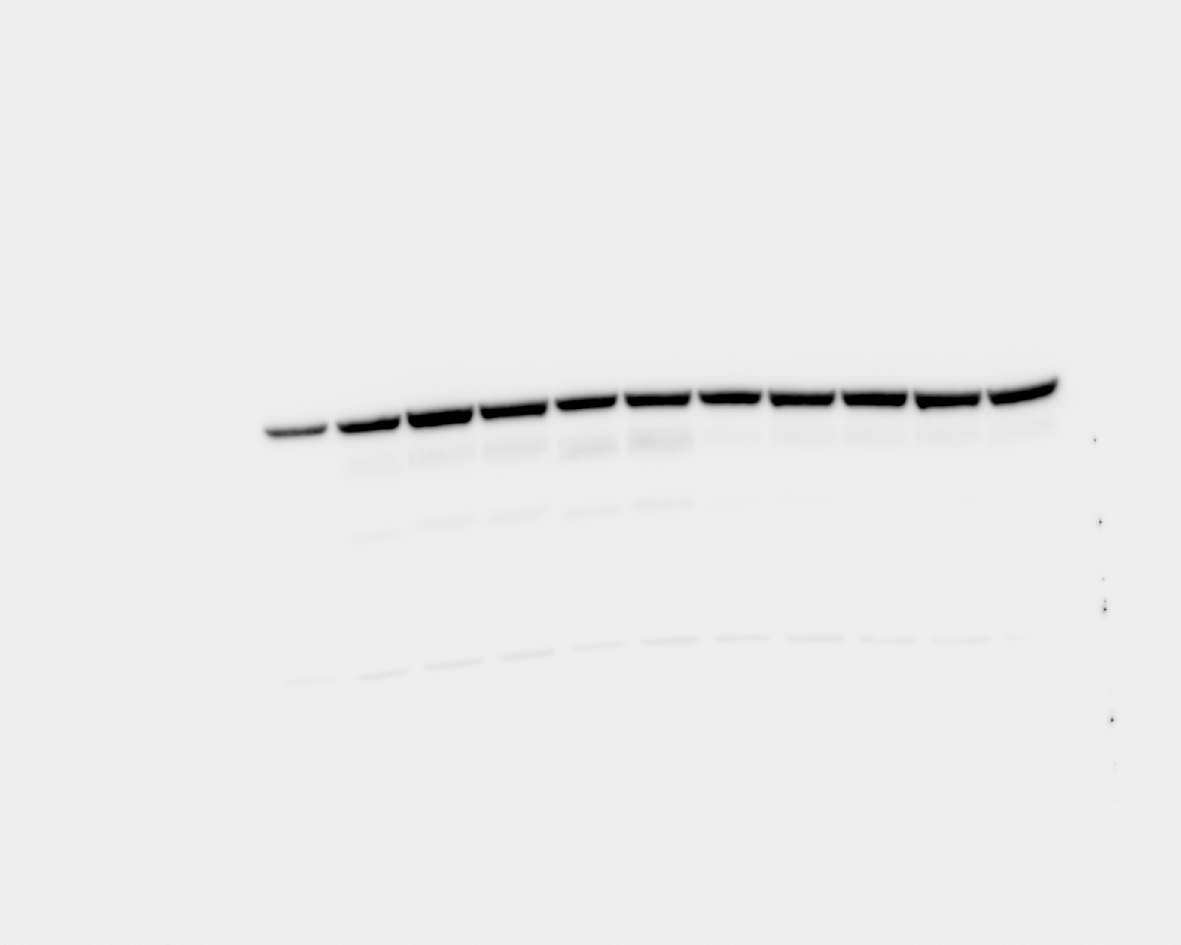

Supplement: Supplementary file 7 — Source Data Fig. 5 [file 44318_2024_32_MOESM7_ESM.zip › Figure 5/Figure 5C Image Data Blot/5C tubulin.tif]

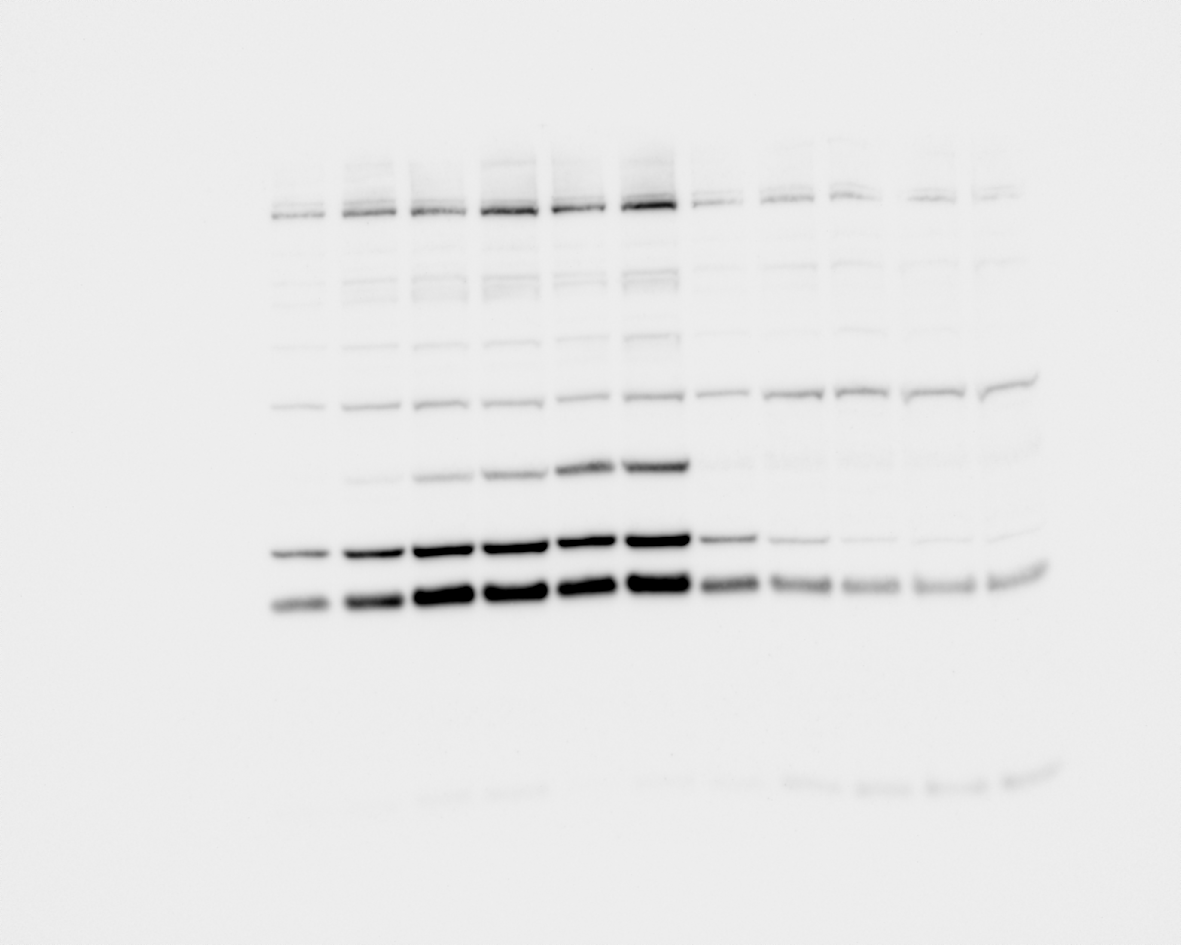

Supplement: Supplementary file 7 — Source Data Fig. 5 [file 44318_2024_32_MOESM7_ESM.zip › Figure 5/Figure 5C Image Data Blot/5C top.tif]
