# Supplementary material for: Qualitative rather than quantitative phosphoregulation shapes the end of meiosis I in budding yeast
Source: EMBO J. 2024 Feb 6;43(7):10. doi: 10.1038/s44318-024-00032-5 (PMC10987528; doi:10.1038/s44318-024-00032-5)
Supplement: Supplementary file 10 — Expanded View Figures [file 44318_2024_32_MOESM10_ESM.pdf]

Expanded View Figures

Figure EV1. The phosphorylation landscape of the MI-MII transition in budding yeast.

(**A**) Protein extracts were prepared at the indicated times and processed for Western blotting against the indicated proteins to monitor synchrony of the pre-meiotic phases. One representative experiment from three independent experiments is shown. (**B**) Percentage of phosphosites in each category of each repeat experiment. (**C**) Overlap between the proteins quantified in the proteome dataset (green) and the phosphoproteome (gray). (**D**) Fractions of proteins identified in the proteome dataset (non-enriched). Protein abundance changes through meiosis I exit are represented in gray (increase), in yellow (decrease) or in black (remain stable). (**E**) Overlap of phosphosites between the core experiment (repeat 1) and the main repeat (repeat 2). Phosphosites are categorized depending on the phosphorylation abundance changes during meiosis I exit - dephosphorylated in red, phosphorylated in blue and remained stable in black. (**F**) Profile plot of the 10 phosphosites dephosphorylated the earliest in the dataset. Timing of anaphase I is indicated. Note that many proteins play a role in the regulation of the synaptonemal complex. (**G**) Profile plot of the 10 phosphosites dephosphorylated the latest in the dataset. Timing of anaphase I is indicated. Note that many proteins are specific and essential for meiotic regulation. (**H**) Profile plot of the 10 phosphosites phosphorylated the earliest in the dataset. Timing of anaphase I is indicated. Note that many proteins are dephosphorylated after being phosphorylated early. Phosphosite intensities are shown on a log scale. (**I**) Profile plot of the 10 phosphosites phosphorylated the latest in the dataset. Timing of anaphase I is indicated. Phosphosite intensities are shown on a log scale.

© The Author(s)

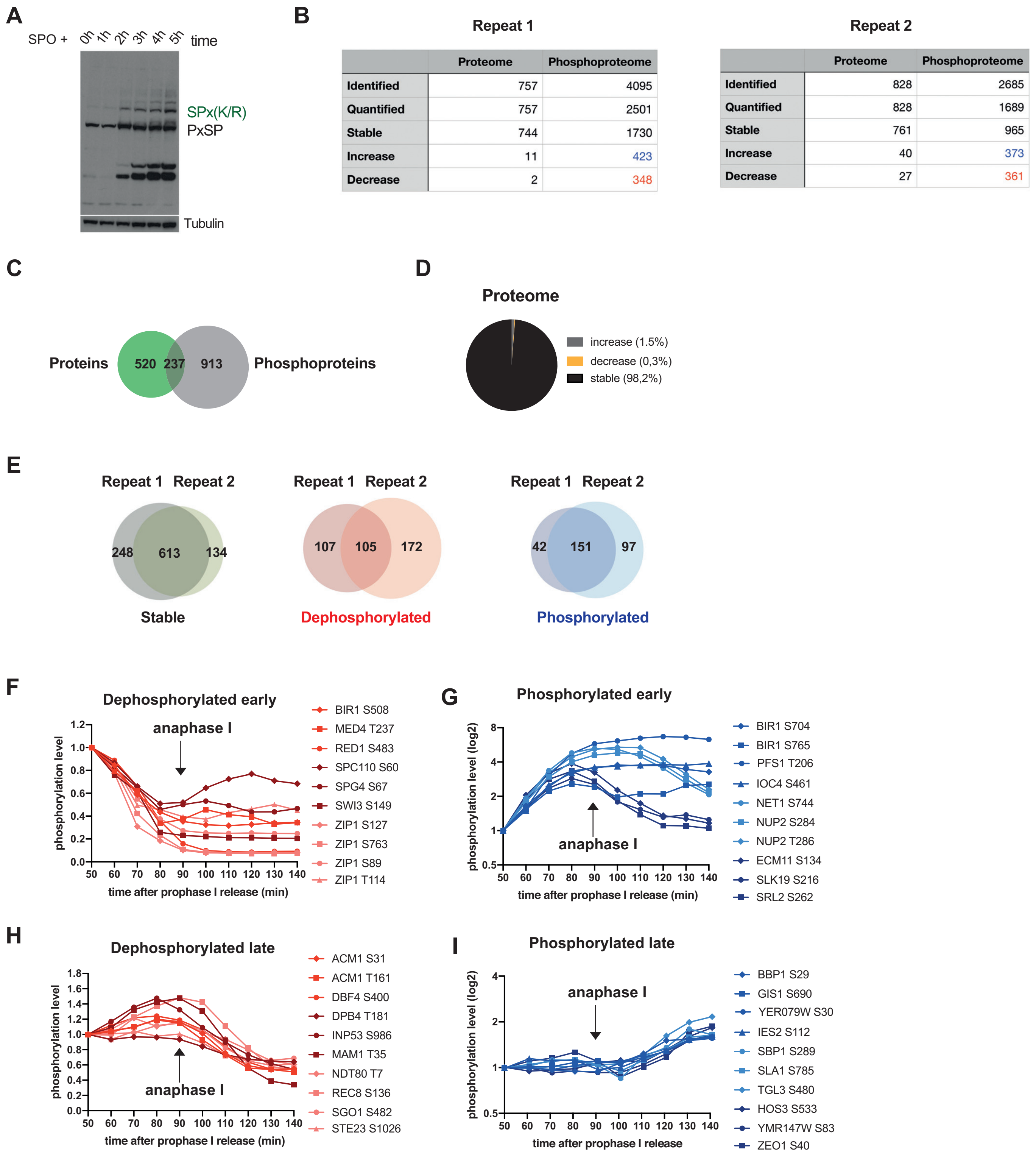
A
SPO +
0h
1h
2h
3h
4h
5h
time
SPx(K/R)
PxSP
Tubulin
B
Repeat 1
	Proteome	Phosphoproteome
Identified	757	4095
Quantified	757	2501
Stable	744	1730
Increase	11	423
Decrease	2	348
Repeat 2
	Proteome	Phosphoproteome
Identified	828	2685
Quantified	828	1689
Stable	761	965
Increase	40	373
Decrease	27	361
C
Proteins
520
237
913
Phosphoproteins
D
Proteome
increase (1.5%)
decrease (0,3%)
stable (98,2%)
E
Repeat 1 Repeat 2
248
613
134
Stable
Repeat 1 Repeat 2
107
105
172
Dephosphorylated
Repeat 1 Repeat 2
42
151
97
Phosphorylated
F
Dephosphorylated early
anaphase I
phosphorylation level
time after prophase I release (min)
BIR1 S508
MED4 T237
RED1 S483
SPC110 S60
SPG4 S67
SWI3 S149
ZIP1 S127
ZIP1 S763
ZIP1 S89
ZIP1 T114
G
Phosphorylated early
anaphase I
phosphorylation level (log2)
time after prophase I release (min)
BIR1 S704
BIR1 S765
PFS1 T206
IOC4 S461
NET1 S744
NUP2 S284
NUP2 T286
ECM11 S134
SLK19 S216
SRL2 S262
H
Dephosphorylated late
anaphase I
phosphorylation level
time after prophase I release (min)
ACM1 S31
ACM1 T161
DBF4 S400
DPB4 T181
INP53 S986
MAM1 T35
NDT80 T7
REC8 S136
SGO1 S482
STE23 S1026
I
Phosphorylated late
anaphase I
phosphorylation level (log2)
time after prophase I release
BBP1 S29
GIS1 S690
YER079W S30
IES2 S112
SBP1 S289
SLA1 S785
TGL3 S480
HOS3 S533
YMR147W S83
ZEO1 S40

© The Author(s)

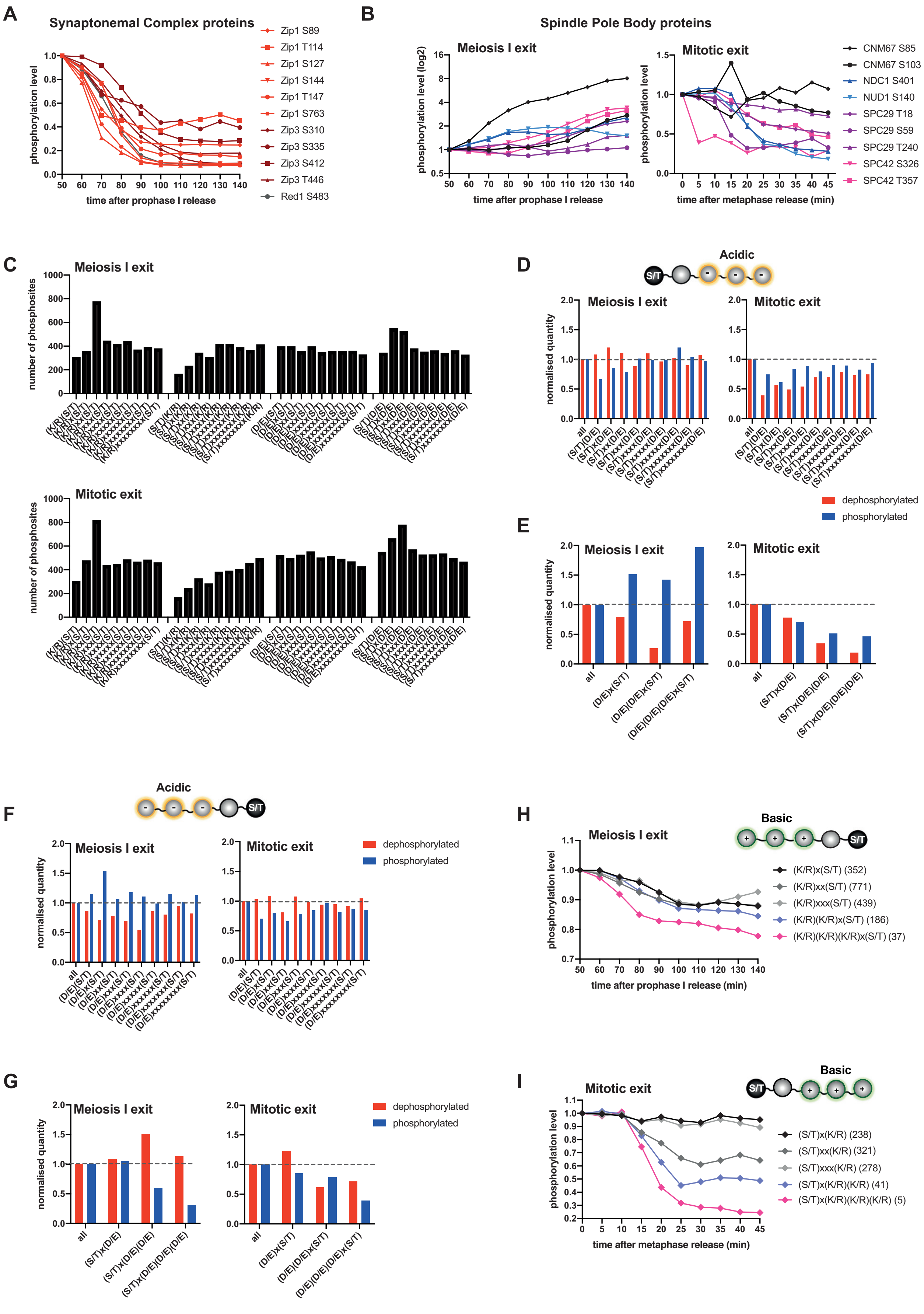
A
Synaptonemal Complex proteins
phosphorylation level
time after prophase I release
Zip1 S89
Zip1 T114
Zip1 S127
Zip1 S144
Zip1 T147
Zip1 S763
Zip3 S310
Zip3 S335
Zip3 S412
Zip3 T446
Red1 S483
B
Spindle Pole Body proteins
Meiosis I exit
phosphorylation level (log2)
time after prophase I release
Mitotic exit
phosphorylation level
time after metaphase release (min)
CNM67 S85
CNM67 S103
NDC1 S401
NUD1 S140
SPC29 T18
SPC29 S59
SPC29 T240
SPC42 S326
SPC42 T357
C
Meiosis I exit
number of phosphosites
Mitotic exit
D
Acidic
Meiosis I exit
normalised quantity
Mitotic exit
dephosphorylated
phosphorylated
E
Meiosis I exit
Mitotic exit
F
Acidic
Meiosis I exit
Mitotic exit
dephosphorylated
phosphorylated
G
Meiosis I exit
Mitotic exit
dephosphorylated
phosphorylated
H
Meiosis I exit
Basic
(K/R)x(S/T) (352)
(K/R)xx(S/T) (771)
(K/R)xxx(S/T) (439)
(K/R)(K/R)x(S/T) (186)
(K/R)(K/R)(K/R)x(S/T) (37)
phosphorylation level
time after prophase I release (min)
I
Mitotic exit
Basic
(S/T)x(K/R) (238)
(S/T)xx(K/R) (321)
(S/T)xxx(K/R) (278)
(S/T)x(K/R)(K/R) (41)
(S/T)x(K/R)(K/R)(K/R) (5)
phosphorylation level
time after metaphase release (min)

Figure EV2. Phosphorylation dynamics of mitotic kinase consensus motifs are reversed at meiosis I exit.

(**A**) Profile plot examples of phosphosites on proteins related to the synaptonemal complex extracted from the meiosis I exit dataset. (**B**) Profile plot examples of phosphosites on proteins related to the spindle pole body extracted from the meiosis I exit dataset (left) and from the mitotic exit dataset (right). Phosphosite intensities are shown on a log scale on the left and on a linear scale on the right. (**C**) Number of phosphosites categorized by amino acid motif identity in the meiosis I exit dataset (above) and the mitotic exit dataset (below). (**D–G**) Percentage of dephosphorylated and phosphorylated sites categorized by amino acid motif identity after normalization to the total amount of sites. Phosphosites with distinct acidic downstream amino acids are presented in (**D**); with single, double, or triple acidic downstream amino acids in (**E**); with distinct acidic upstream amino acids in (**F**); and with single, double, or triple acidic upstream amino acids in (**G**). (**H**,**I**) Median intensity profile of the phosphosites categorized by amino acid motif identity through meiosis I exit. Phosphosites with basic upstream amino acids are presented in (**H**) and with basic downstream amino acids are presented in (**I**).

© The Author(s)

A

Mitotic exit \ Meiosis I exit	Phosphorylated	Stable	Dephosphorylated
Phosphorylated	4	39	13
Stable	43	401	93
Dephosphorylated	39 (3)	86 (2)	9 (1)

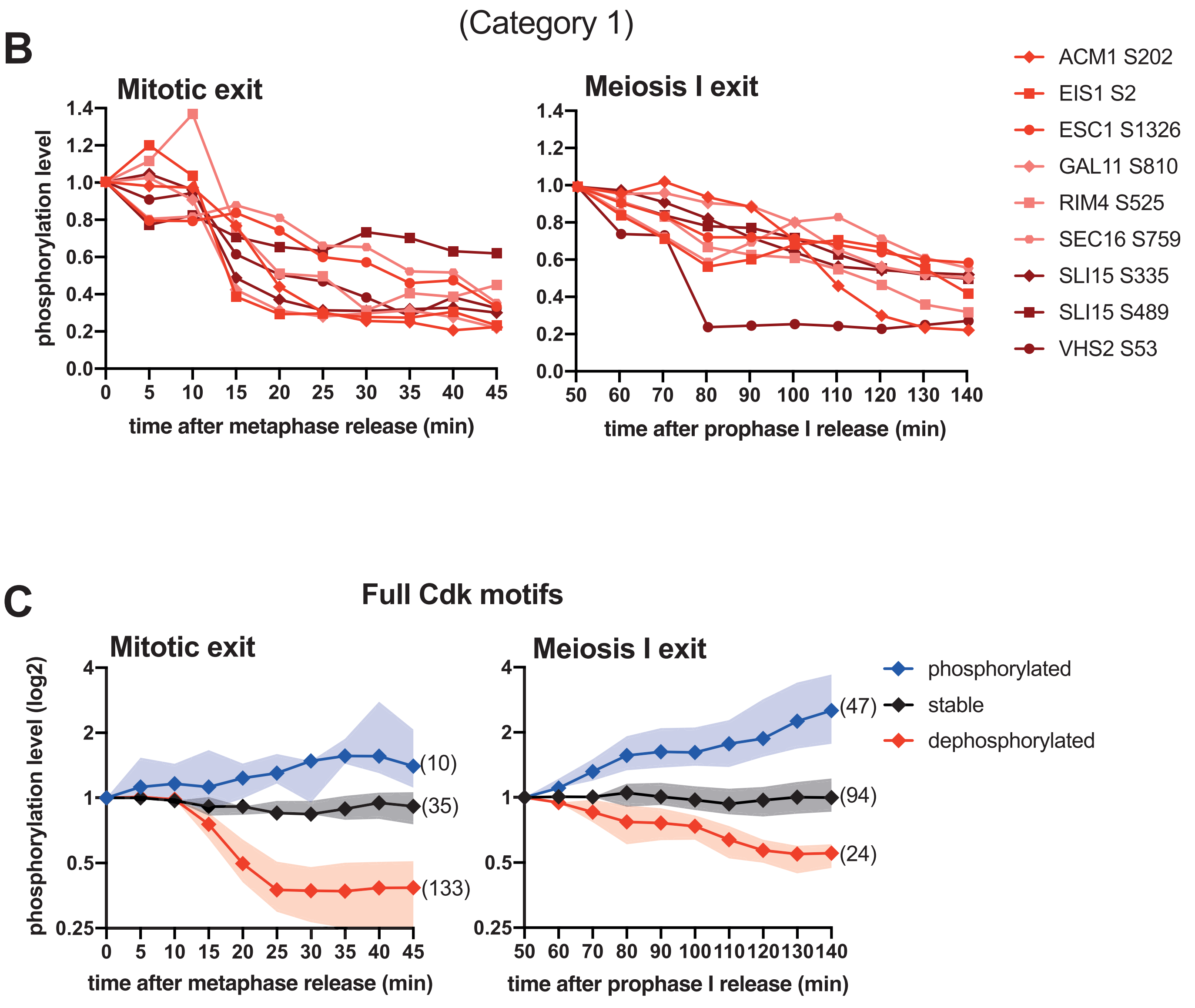

Figure EV3. Full Cdk sites dephosphorylated during mitotic exit are mostly stably phosphorylated during meiosis I exit.

(**A**) Table showing the number of phosphosites that are in common between the mitotic exit dataset and the meiosis I exit dataset in each category. (**B**) Profile plot of the 9 phosphosites dephosphorylated in both the mitotic exit and meiosis I exit dataset. (**C**) Normalized median intensity profiles and interquartile range of the full Cdk sites (S/T)Px(K/R) that undergo a 1.5-fold decrease (red) or a 1.5-fold increase (blue) in phosphorylation abundance through mitotic exit (left) and meiosis I exit (right). Normalized median intensity profiles and interquartile range of the phosphosites remaining stable are in black. Phosphosite intensities are shown on a log scale.

© The Author(s)

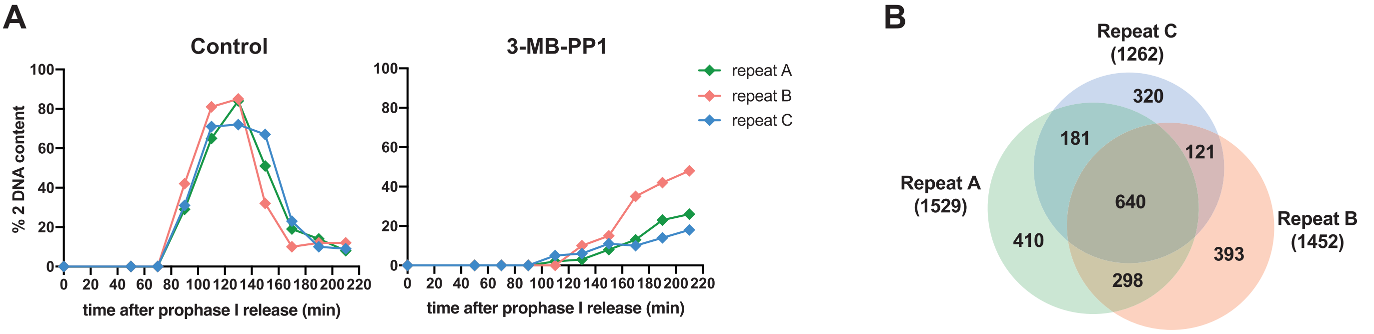

C

	Repeat A	Repeat B	Repeat C
**Decrease**	29,62 %	17,42 %	10,38 %
**Increase**	32,57 %	22,03 %	3,32 %

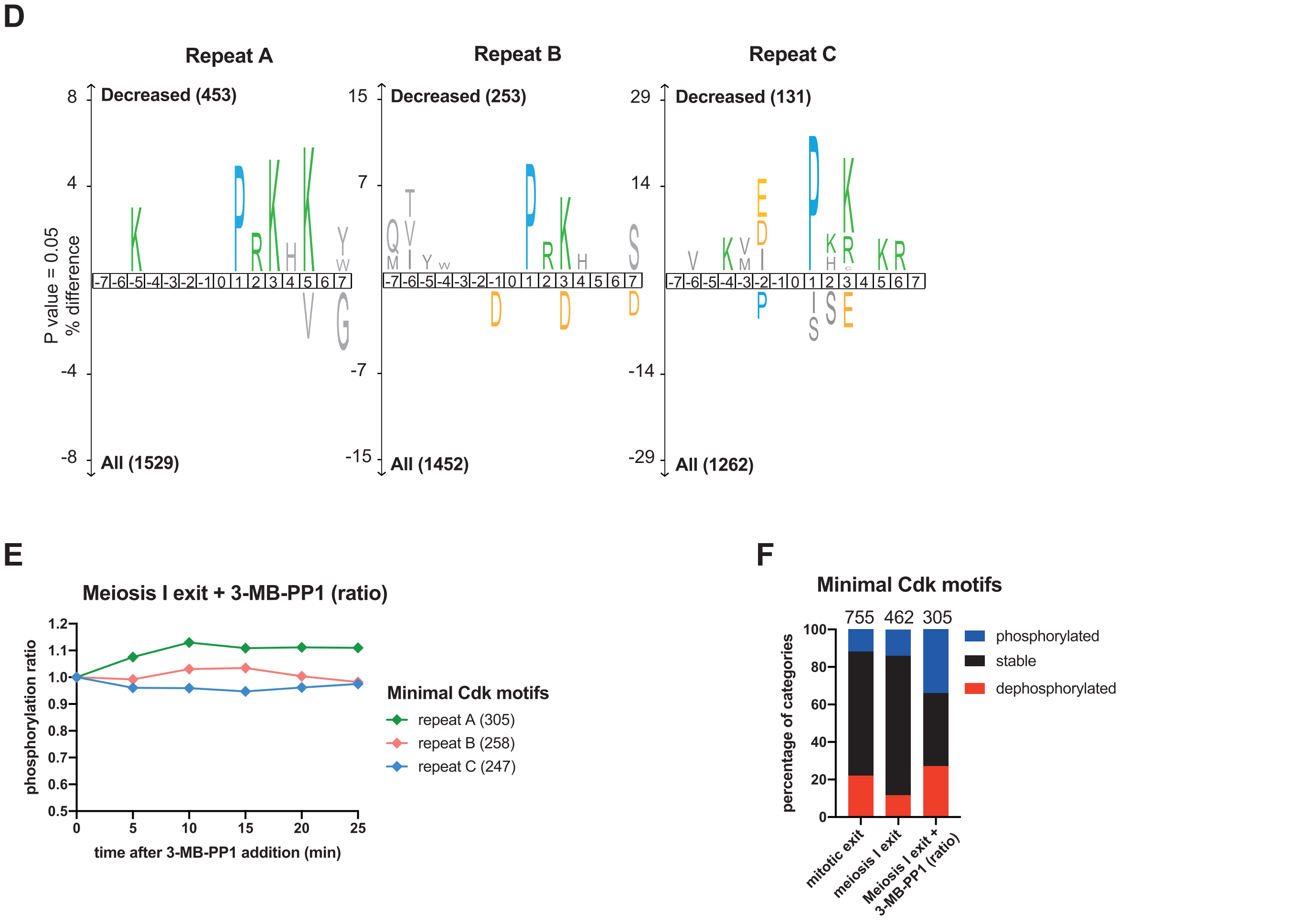

 © The Author(s)

◀ **Figure EV4. Phosphorylation landscape at meiosis I exit after complete drop in Cdk activity.**

(**A**) One hundred cells were scored for 2 DNA content at each timepoint (0 to 220 min after β-estradiol addition) in absence or presence of 3-MB-PP1 to determine the cell cycle phases. The timing for the three repeats is presented. (**B**) Overlap of phosphosites between the three repeats. (**C**) Table showing the percentage of phosphosites decreased or increased in each repeat. (**D**) IceLogo analysis in the three repeats highlighting phosphomotifs where phosphorylation abundance decreases after addition of 3MB-PP1. The phosphorylated residue is at position 0. Larger letter size indicates increased enrichment. Percentage of difference is used as scoring method. The threshold for enrichment detection was $p = 0.05$. (**E**) Median intensity profile of sites phosphorylated on the minimal Cdk motifs in the 3 repeats. Ratio values are plotted. (**F**) Percentage of the minimal Cdk sites (S/T)P in each category. Of note, this category excludes full Cdk sites (S/T)Px(K/R).

© The Author(s)

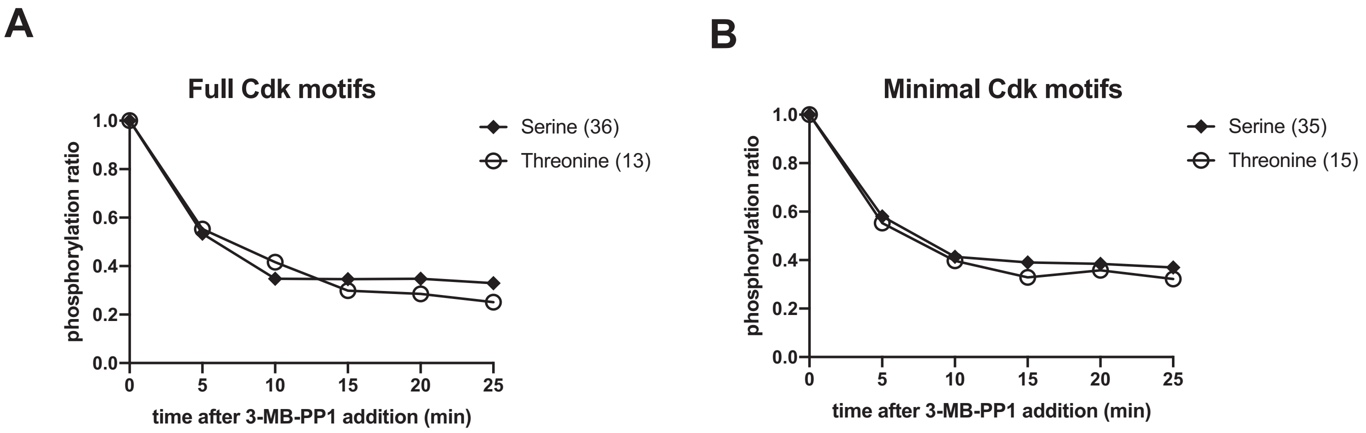

Figure EV5. Certain phosphorylation patterns at meiosis I exit remain meiosis-specific despite Cdk inhibition.

(**A**,**B**) Normalized median intensity profiles of the phosphosites that decrease and adhere to full Cdk motifs (**A**) or minimal Cdk motifs (**B**).

 © The Author(s)
